# Research on affective cognitive education and teacher–student relationship based on deep neural network

**DOI:** 10.3389/fpsyg.2022.991213

**Published:** 2022-09-28

**Authors:** Shi Zhou

**Affiliations:** School of Marxism, Anhui Medical University, Hefei, China

**Keywords:** neural networks, emotional cognition education, teacher–student relationship, neural network, research

## Abstract

Since entering the new century, People’s living standards are constantly improving, with the continuous improvement of living conditions, people are becoming more and more important in education, which is the embodiment of the enhancement of national strength. The education level is getting higher and higher, and a good education level needs a good teacher–student relationship. To solve these problems, we use the emotional cognition of God’s network to study the teacher–student relationship, and collect and analyze the data of the teacher–student relationship. In this chapter, we use GABP neural network algorithm DHNN algorithm and discrete Hopfield neural network to make the collected data more convenient to be analyzed. The research shows that there is a close relationship between the educational level and the relationship between teachers and students in China, and a good relationship between teachers and students will promote the improvement of educational level. According to the research data, “face-to-face” is the most important way of interaction between tutors and postgraduates in various types of teacher–student relationship. QQ WeChat is also one of the main ways of interaction between students and teachers, which shows that the interaction between students and teachers is talking about the interaction between online and Internet. The education industry is becoming more and more important, and the teacher–student relationship is the most important part of the education industry. Good teacher–student relationship is helpful to cultivate students’ healthy personality. In view of the cold relationship between teachers and students at present, we need to make some measures the relationship between teachers and students and effectively use the relationship between teachers and students to promote the better development of the education industry.

## Introduction

This method of neural network self-adaptive generation of small fluctuations for signal representation and classification. Different network structures and energy functions are necessary, and the classification model is given. One-dimensional signal is simulated, and the concept is extended to image. The idea of applying the concepts in this paper to phoneme and speaker recognition is discussed (Szu, 1992). In this paper, we study the impulse effect of a class of impulsive n-dimensional neural networks with unbounded delay and supermom on global exponential stability. We use Lyapunov function method to build a stable model. Three illustrative examples are given to prove the validity of the obtained results. This technique can be extended to study multidimensional delay systems of other impulses (Stamova et al., 2014). The network based on Takagi Sugeno model consists of two parts: premise network and follow-up network. The proposed network has the ability of local mapping, which shows the advantages of neural network and fuzzy logic. Networks can easily express vague and qualitative knowledge. It also has good learning ability (Sun and Xu, 1997). The purpose of this work is to define a program that uses wavelet transform and neural network to process the generated image to achieve lower computational cost and acceptable accuracy. Wavelet space represents compact support for efficient feature extraction with location characteristics. The proposed solution is demonstrated on several defects of different types of circuits (Belbachir et al., 2005). In this paper, the preliminary results of a study are given to determine the influence of noise training set on fault tolerance. Back propagation is used to train three networks in 7 × 7 digital mode. One network controls and uses noise-free inputs, and the other two networks use two different noise conditions. The network trained on noisy input has better fault tolerance than the network trained on noiseless input (Minnix, 1992). Describe psychological mechanisms, explain how emotions affect thoughts, from daily decisions to scientific discoveries and religious beliefs, and analyze when emotions contribute to good reasoning (Thagard, 2006). In this paper, several authors describe various skills needed to develop emotional ability, emotional self-efficacy and even mental health in social situations (Nader-Grosbois and Day, 2011). This is a comparative study. Through the task of distinguishing emotional expression, the results show that the ability to distinguish emotional expression of NLD children, VLD children and normal children develop as they grow up, but the ability development of NLD children is slower than that of the other two groups. Children in the National League for Democracy are more sensitive to negative expressions such as sadness, fear and anger, and it is difficult for them to distinguish complex expressions such as disgust (Zhang, 2008). Traditional rationalism treats cognition and emotion separately. Pre-service teachers’ emotional experiences in teacher preparation courses are preparing courseware articles on emotional cognition (Yang, 2019). Affective cognitive modeling is still in its infancy, so it is open to new ideas and discussions. In this paper, the author studies the modeling problem by introducing the ideas of axiomatic mathematics, information theory, computer science, molecular biology, nonlinear dynamic system and quantum computing, and explains how these ideas are applied in emotional cognitive modeling (Bera, 2013). This paper integrates the results of three independent studies (one national quantitative study and two urban qualitative studies), and analyzes two aspects of teacher–student relationship: (a) how teachers and students view their relationship, and (b) how this relationship affects students’ subsequent academic achievements. These three studies all confirm an important discovery, that is, teachers’ educational expectations are mainly based on students’ test scores, while students’ shaping of their educational expectations mainly depends on their views on teachers’ expectations and their test scores (Muller et al., 1999). Affective factors play an increasingly important role in foreign language teaching. Teacher–student relationship is one of them. This paper attempts to find out the existing relationship between teachers and students in college English teaching through relevant investigations. Some suggestions on how to establish a harmonious relationship between English teachers and students are put forward (Xiang, 2004).This paper studies the influence of teacher–student warmth and conflict on children who dislike and like peer nominations of children in grades 1–4 (6–10 years old). Multi-level modeling controls the time-invariant differences among children, and simulates the influence of time-varying teacher–student relationship (TSR) warmth and conflict on children’s peer correlation. Teachers reported warmth and conflict (Hughes and Hee Im, 2016). This is a descriptive study of teachers’ views on establishing and maintaining a positive teacher–student relationship at the undergraduate level. This study is a qualitative study involving interviews (Hussain, 2014). Based on the experience of science and mathematics curriculum in primary and secondary schools, this paper discusses the changes of classroom dynamics when computer technology is included in the curriculum. Topics include student motivation; Cooperation and collaboration; Increase perseverance in solving problems; Different evaluation methods; Increase and improve communication; and interdisciplinary opportunities (Mcgrath, 1998).

## The relationship between teachers and students

### Importance of teacher–student relationship

In today’s social education system, the relationship between teachers and students is not only “teaching” and “learning,” but also the intimate relationship between friends. Only in this way can students grow up in a perfect teaching environment, which is a new type of teacher–student relationship. A good teaching environment is the most basic requirement for students to receive education, in addition to complete and advanced teaching facilities, whether the relationship between teachers and students is harmonious or not is also the most important thing. Only when teachers and students have the same heart, respect and trust each other, and have a spiritual bridge across each other, can they create a harmonious classroom together and let teachers better teach their knowledge and skills to students without room. Good teacher–student relationship is helpful to further strengthen students’ practical innovation ability. Contemporary college students generally have a strong concept of equality and democracy, and hope that teachers fully respect individual rights. If students are allowed to express their personal wishes and demands in an equal and democratic environment, they can effectively enhance their enthusiasm and help cultivate and show their creative ability and innovative ability. The education industry is becoming more and more important, and the teacher–student relationship is the most important part of the education industry. Good teacher–student relationship is helpful to cultivate students’ healthy personality. In view of the cold relationship between teachers and students at present, we need to make some measures to promote the relationship between teachers and students, and effectively use the relationship between teachers and students to promote the better development of the education industry.

### How to deal with the teacher–student relationship

The purpose of implementing the new curriculum standard is to embody the curriculum reform idea of “taking people as the foundation of development,” which provides a platform for teachers and students to develop together. In the situation of equal treatment between teachers and students, teachers and students face not only knowledge and teaching materials, but also a wider range of real life. Therefore, teachers and students need to communicate information when there are cognitive differences in teaching content, learning spirit, behavior concept and other places. In the process of teaching experiment, because teachers and students play different roles in different positions, some contradictions are certain. How to solve these contradictions requires teachers and students to consciously use some means to adjust. As the driving force of education, Teachers should first correct their attitude, Enthusiastic care for every student, students with psychological problems should learn to be patient, calm, fair and reasonable, not serious about students’ mistakes, but only solve things on the external performance. In order to avoid one-sided injustice, some students are punished, which will make the contradiction between teachers and students deeper and deeper, which will lead to the failure of education. In addition, there are some teachers who do not seriously reflect on their own mistakes and punish students. Such teachers make students dissatisfied and will not produce good teaching results. On the contrary, a teacher who likes to reflect on himself and deal with his relationship based on the principle of thinking for others will increase students’ goodwill toward teachers and students will have great interest in class.

### Present situation of university teacher–student relationship

Teachers and classmates are the two largest groups in the school. Students are in a very important period of learning and understanding knowledge, half into the society and learning life. Dealing with the relationship between teachers and classmates has a great relationship with their learning and growth, as well as their interpersonal relationships and career success after entering the society in the future. However, in many schools, the relationship between most teachers and students is not very good, and sometimes they do not talk like this when they meet, just like they do not know each other. Our survey of students in three schools shows that 4.0% of students think that the relationship between teachers and students is “good,” 17.2% think that the relationship between teachers and students is “better,” 59.6% think that the relationship between teachers and students is “not very good,” and 19.1% think that the relationship between teachers and students is “very cold.” According to other surveys, 74.1% of college students think that the teacher–student relationship is “not very good,” and 11% think that it is “very bad.” Therefore, many teachers and students think that the relationship between teachers and students in schools is “not very good,” even “very bad.” On the whole, it is normal for teachers and students to “respect teachers and love children” on campus. Most students still respect teachers, and teachers can treat and care for students equally. However, the number of exchanges between teachers and students is not high, and the relationship between teachers and students is not good enough. This situation is something we need to consider.

### Requirements of good teacher–student relationship for teachers

A good teacher–student relationship needs a good teacher relationship to standardize the constraints and. Teachers’ ideas and behaviors have great influence on students’ behaviors. Therefore, college teachers should strictly abide by the norms of teachers’ morality. Love students, treat students equally. Teachers’ love for students, It is a feeling formed by teachers in the process of performing their duties. It is not based on blood relationship, nor is it based on personal interests or preferences, but for the actual interests of education itself and students. It comes from teachers’ love for their profession and strong sense of responsibility. Teachers’ love should face all students, especially those “underachievers,” who should not be abandoned and bored, and should always hold a strong sense of responsibility to guide them to make continuous progress. Only when teachers have a comprehensive understanding of students’ advantages and disadvantages, can they explore their bright spots and valuable points, combine students’ respective characteristics, and seek educational methods that are consistent with students’ personal development needs, so as to truly teach students in accordance with their aptitude, stimulate each student’s potential to the greatest extent, and enhance their learning enthusiasm.

## Overview of neural network algorithms

### GABP neural network algorithm

It is a combination of GABP (GA) and BP. The GA algorithm is an artificial algorithm. Using useful mathematical techniques, this operation changes the answer to several questions about genes in biological processes.

GABP is the combination of (GA) and BP. GA algorithm is a kind of algorithm summarized and put forward by people. Through useful mathematical methods, it carries out operations and turns the answers to some questions into some genes in biology. GA can get results quickly when combining, so it is applied in the fields of problem solving and information collection. BP network is based on the existing algorithm of BP network, arbitrarily select a group of data, give specific target data directly as the numerical value in the equation, create the equation group, and get the result by solving this equation, which can solve the problems of slow speed and local minimum of traditional methods. The training of BP network can be divided into four matrix methods. Where the input data of the matrix is W, the hidden data is Y, and the obtained data is H, the numerical matrix from the input layer to output layers is shown in the following formula.

This article collected a large number of data through the comparison between charts to deal with the collection, the establishment of mathematical model makes the whole paper more rigorous. Chart information makes the conclusion of the whole paper more reliable and accurate, and the establishment of functions and mathematical models can reflect the development trend of the experiment.


(1) $$W=\left[\begin{array}{l} W_{11}W_{12}\ldots W_{ij}\\ W_{21}W_{22}\ldots W_{2j}\\ \ldots \\ W_{i1}W_{i2}\ldots W_{ij} \end{array}\right]$$

The hidden layer threshold matrix is:


(2) $$Y=\left[\begin{array}{c} y_{1}\\ y_{2}\\ \cdots \\ y_{j} \end{array}\right]$$

The weight matrix from hidden layer to output layer is:


(3) $$V=\left[\begin{array}{c} V_{11}V_{12}\cdots V_{1K}\\ V_{21}V_{22}\cdots V_{2K}\\ \cdots \\ V_{j1}V_{J2}\cdots V_{JK} \end{array}\right]$$

The threshold matrix of the output layer is:


(4) $$H=\left[\begin{array}{c} H_{1}\\ H_{2}\\ \cdots \\ H_{K} \end{array}\right]$$

Using GA algorithm, solve the BP network processing, the process of solution can be run through the above algorithm. When designing GABP network algorithm, we need to focus on these aspects: First, input. In fact, the input module is not only responsible for data input, Preliminary data processing is also needed, It can be divided into two sub-modules. In the data input module, it is necessary to determine the initial indicators and assign them to the selected system. Combined with the actual needs of the security system and relevant policies and regulations, the index system set in the initial state is defined, and the data acquisition system is constructed according to the formed system set. Secondly, the operation module. The operation module can also be divided into two sub-modules. One is the index analysis module, which can analyze from the perspective of data indicators and reflect the relationship between data and indicators. With this relationship, we can analyze the characteristics of index data and study the combination characteristics of index groups; The second is the evaluation model data module, which can analyze the network security, find the security problems existing in the system, and put forward corresponding solutions. Then there is the output module. In the complex computer network security evaluation system, the output module needs to include multiple target sub-modules: first, the security monitoring module, which can evaluate the current security state of the computer network; The second is the security early warning module, which can carry out early warning processing at any time from the actual situation of the computer network and prompt and alarm possible security problems; The third is the safety control module, which can judge the sent safety early warning information and realize management control in combination with the standardized safety indicators, so as to ensure the safety and stability of the system operation; The fourth is the visual display module, which can visually display the security problems existing in the system with the help of charts and other forms. Finally, qualitative and quantitative. In the data input link, with the help of the corresponding parameter operation, the evolution form of the security system is analyzed, and the parameters with different changing rules are obtained through the corresponding analysis mode, and they are classified and evaluated. In the process of classification evaluation, various qualitative and quantitative problems will be caused because of the influence of different factors. To solve these problems, we must pay attention to the types of problems. At present, neural network is BP network in solving complex problems, has a very big role.

### Discrete Hopfield neural network

Discrete Hopfield Network is a special network with N neuron nodes. The output of each neuron can be connected to the received data of other neurons. Each node has no self-feedback, and each node can be in a possible state (1 or −1), that is, when the stimulus received by the neuron exceeds its threshold, the neuron is in an excited state (such as 1), otherwise the neuron is always in an inhibited state (such as −1). For a network with symbolic function as the key.

Hopfield Neural Network combines some important mathematical formulas to make the network system more stable and the results are more accurate.


(5) $$u_{i}\left(t+1\right)=\overset{n}{\underset{j=1}{\sum }}w_{ij}x_{j}\left(t\right)$$

Formula (5) uses a special vector to connect the data matrix, and its working mode changes its working state according to Formula (5) at a specific time, which makes the working state more perfect and Formula (5) can solve more complex network problems.


(6) $$x_{i}\left(t+1\right)=\mathrm{sgn}\left[u_{i}\left(t\right)\right],\left(i=1,2,\cdots ,n\right)$$

$W={\left(w_{ij}\right)}_{n\times n}$ to join the data matrix, θ for special vectors, the briefly described H=(W,θ). At a certain time, its working (5), output methods of other neurons remain unchanged. This data can be randomly selected (6). In the parallel operation mode, N digits change state according to formula (5) at a certain time, while the output of other neurons remains unchanged. The changing group of neurons You can choose in different ways. When N = N, it is called complete mode. If you can pass formula (7)


(7) $$x_{i}\left(t+1\right)=x_{i}\left(t\right)=\mathrm{sgn}\left(\overset{n}{\underset{j=1}{\sum }}w_{ij}x_{i}\left(t\right)-\theta_{i}\right),i=1,2,\cdots ,n$$

X is the data point. For the analysis of network stability, there are the following theorems: When the network state is adjusted according to the network state, synchronously and the Matrix W is the same matrix for all computers H=(W,θ) will receive 1 special data or 1 special ring with a length of 2.

### DHNN algorithm

In order to avoid solving the concrete solution of continuous neural network, and only solve the classification problem of complex network by symbols, this paper proposes a new algorithm for extracting community structure of complex structure algorithm: DHNN algorithm for extracting community structure based on neural network. The main ideas of the discrete algorithm are as follows: Firstly, the algorithm still takes the modularity function Q as the objective function. Secondly, the continuous differential equation is changed:


(8) $$\frac{dX\left(t\right)}{dt}=AX\left(t\right)-X^{T}\left(t\right)AX\left(t\right)X\left(t\right),\left(A^{T}=A\right)$$

Discrete Hopfield network data are obtained by decentralized processing. Finally, according to the stable point data of A decentralized model is proposed to transform difficult networks into two categories. A difficult network with *n* pieces of data, it is usually used N=(V,E), then the corresponding network of *n* data can be obtainedH=(W,θ). The value W represents a special matrix, θ represents a corresponding vector, and this vector $X={\left(x_{1,\cdots }x_{n}\right)}^{T}\in +{\left[+1,-1\right]}^{n}$. Function of data $x_{i}\to f\left(v_{i}\right)$, $v_{i}$ is the survival state of the letter I:


(9) $$v_{i}\left(t\right)=\overset{n}{\underset{j=1}{\sum }}w_{ij}x_{j}\left(t\right)-\theta_{i}$$

In asynchronous mode, when only one data state changes at a time, the selection of I can be changed at any time. Specific changes are shown in Equations 10 and 11.


(10) $$\begin{array}{l} x_{i}\left(t\right)=f\left(v_{i}\left(t\right)\right),v_{i}\left(t\right)=\overset{n}{\underset{j=1}{\sum }}w_{ij}x_{j}\left(t\right)-\theta_{i}\\ x_{j}\left(t+1\right)=x_{j}\left(t\right),j\neq i \end{array}$$

In the case of synchronization:


(11) x(t+1)=f(v(t)),v(t)=Wx(t)−θ

F is a special formula, and all *n* data in this paper work together, so discrete sign function is taken:


(12) $$f\left(v\right)=sign\left(v\right)=\left\{\begin{array}{l} +1,v\geq 0\\ -1,otherwise \end{array}\right\}$$

In this way, you can get:


(13) $$\begin{array}{l} x\left(t+1\right)=sign\left(Wx\left(t\right)-\theta \right)\\ x_{i}\left(t+1\right)=x_{i}\left(t\right)=sign\left(w_{ij}x_{j}\left(t\right)-\theta_{i}\right),i=1,2\cdots ,n \end{array}$$

When this was set up, all the networks stabilized. For formula (13), the continuous differential equation is written in the form of difference, and you can get:


(14) $$\begin{array}{l} \frac{x\left(t+1\right)-x\left(t\right)}{\left(t+1\right)-t}=Bx\left(t\right)-x^{T}\left(T\right)Bx\left(t\right)x\left(t\right)\\ x\left(t+1\right)=Bx\left(t\right)+x\left(t\right)-x^{T}\left(t\right)Bx\left(t\right)x\left(t\right) \end{array}$$

By combining (13) with (14), a discrete network model:


(15) $$x\left(t+1\right)=sign\left(Bx\left(t\right)+x\left(t\right)-x^{T}\left(t\right)Bx\left(t\right)x\left(t\right)\right)$$

For dispersed Hopfield networks H=(W,θ). Take $\theta =x\left(t\right)-x^{T}\left(t\right)Bx\left(t\right)x\left(t\right)=0$changed to:


(16) x(t+1)=sign(Bx(t))

The modularity matrix B in formula (16) disassembled to obtain:


(17) $$X\left(t+1\right)=sign\left(BX\left(t\right)\right)=sign\left(AX\left(T\right)-\frac{k_{i}k_{j}X\left(t\right)}{2m}\right)$$

Where A represents the *n* × *n* matrix of a particular network. X (t) denotes an *n* special vector with a value of ±1. kI is a value of data i, signed function


(18) $$sign\left(x\right)=\left\{\begin{array}{l} 1,x\geq 0\\ -1,,x∠0 \end{array}\right\}$$

In the problem of extracting Complex network, numbers are investigated by our structure. For the i-th neuron, let $x_{i}=\{\begin{array}{l} +1,i\in S\\ -1,i\notin S \end{array}$,$x\in {\left\{+1,-1\right\}}^{n}$ It represents a state, a special subset S of the original data set V. When the (16) when the network is stable, the symbols of the network data can be divided into two categories in the corresponding output.

### Stability analysis of 3DHNN algorithm

The stability of discrete Hopfield neural network represented by formula (16) can be analyzed by its energy function. Discrete Hopfield neural network H=(W,θ) the energy function is defined as follows:


(19) $$E=-\frac{1}{2}\overset{n}{\underset{i=1}{\sum }}\overset{n}{\underset{j=1}{\sum }}w_{ij}x_{i}x_{j}+\overset{n}{\underset{i=1}{\sum }}\theta_{i}x_{i}=-\frac{1}{2}X^{T}WT+X^{T}\theta$$

The modularity function Q proposed by Newman is shown in (13). Because the modularity matrix B of Q is symmetric, W in (19) is taken as B, θ=0. Then, formula (19) can be expressed:


(20) $$E=-\frac{1}{2}\overset{n}{\underset{i=1}{\sum }}\overset{n}{\underset{j=1}{\sum }}w_{ij}x_{i}x_{j}+\overset{n}{\underset{i=1}{\sum }}\theta_{i}x_{i}=-\frac{1}{2}X^{T}BX=-Q$$

The expression for the function Q is of the same form as the one that defines the energy function of the Hopfield network, but with the opposite sign. If the value of the modular function Q is used to measure the quality of the community structure, the maximum value of Q and the Hopfield neural network are required.

That is to say, the expression of modularity function Q is the same as the definition expression of energy function of Hopfield network, and their symbols are opposite. When the value of modularity function Q is used as the measure of community structure extraction, the maximum value of Q is required, and just in Hopfield neural network. In order to get the stable point of network state, the minimum point of energy function needs to be obtained, because the minimum point of energy function corresponds to the stable point of network. Therefore, when the stable state of the dispersed network is obtained, and the maximum value of the model is obtained, and a partition of complex network nodes can be obtained according to the symbols of elements in the stable point of discrete Hopfield neural network.

## Teacher–student relationship research

### Research on the relationship between teachers and students on students’ mental health

A 2*4 analysis of variance was conducted with teacher–student relationship as dependent variable and children’s sex and age (T1) as independent variables. The results showed that sex (*f* = 0. 40, *p* > 0.05), age (f = 0. 09, *p* > 0.05) and the interaction between them (f = 0. 84, *p* > 0.05) were not significant. This result shows that the teacher–student relationship of migrant students is not different because of the difference of students’ gender and age. The children’s depression in T1 and T2 is the dependent variable, and the students’ gender and age are the independent variables. The variance analysis of 2*4 is carried out. The results showed that the sex (Fs < 2.59, ps > 0.05), age (Fs < 1.54, ps > 0.05) and the interaction (Fs < 1.55, ps > 0.05) of depression in T1 and T2 students were not significant. This result shows that the anxiety and depression levels of migrant students are not different due to gender and age. From the data, we get the following chart 1. By analyzing Table 1, we can find that the teacher–student relationship in T1 is negatively correlated with the anxiety of children in T2 and T3, which shows that the better the relationship between teachers and students, the lower the anxiety level of students. The results showed that the sex (Fs < 2.59, ps > 0.05), age (Fs < 1.54, ps > 0.05) and the interaction between them were not obvious in T1 and T2 students. This result shows that students’ anxiety and depression levels are not different due to gender and age differences.

**Table 1:** The relationship between teacher–student relationship and children’s mental health.

	1	2	3	4	5
1t1Teacher–student relationship	1	10	0	0	0
2t1Students’anxiety	−0.18^**^	1	0	0	0
3t2Students’anxiety	−0.47^**^	0.58^**^	1	0	0
4t1Studentdepression	−0.18^**^	0.67^**^	0.55^**^	1	0
5t2Studentdepression	−0.34^**^	0.48^**^	0.70^**^	0.61^**^	1

** *P* < 0.01.

By analyzing Table 1, it is found that the teacher–student relationship, anxiety and depression of migrant children are analyzed by Pearson product-difference correlation analysis. The results (see Table 1) show that the teacher–student relationship in T1 is negatively correlated with anxiety and depression of children in T1 and T2, and there is a significant positive correlation between anxiety of children in T1 and T2 and depression of students in T1 and T2. This result shows that the better the teacher–student relationship, the lower the immediate anxiety and depression level of students, and the lower the anxiety and depression level of students after half a year.

Descriptive statistics and correlation analysis were made by using the average scores of teacher–student relationship questionnaire, mathematics anxiety questionnaire and perceived teachers’ cognitive stimulation strategy questionnaire. The statistical results of data and related data results are shown in Table 2. The results showed that teacher–student relationship, perceived teacher’s cognitive stimulation strategies and high-level mathematics ability were positively correlated with each other, with a correlation between 0.229 and 0.362. The correlation coefficients of teacher–student relationship and teacher’s cognition were −0.205, −0.117, and −0.374, respectively. The particularity of each variable was more obvious at 0.01.

**Table 2:** Descriptive statistics and results analysis of each research variable (*N* = 5,247).

Variable	M + SD	1	2	3	4
Variable	3.55 + 1.00	1			
Mathematics anxiety	2.67 + 1.00	−0.205^**^	1		
Perceived cognitive stimulation of teachers	3.48 + 0.77	0.362^**^	−0.117^**^	1	
High-level mathematical ability	44.41 + 26.94	0.229^**^	−0.374^**^	0.259^**^	1

** *P* < 0.01.

The researchers tested the mediating effect through the deviation-corrected nonparametric percentile Bootstrap method. In the long run, 1,000 Bootstrap data were selected from the number (*N* = 5,247) by sampling method, and 95% confidence interval was calculated. It is concluded that 95% interval of path coefficient includes 0, indicating that the mediating effect is significant. Table 3 shows the relationship between teachers and classmates and the result analysis of mathematical ability. Among them, the mediating effect of teachers’ cognitive stimulation strategies perceived by students is the strongest, the mediating effect is 0.09 [0.07, 0.11], the mediating effect of mathematics anxiety between teacher–student relationship and high-level mathematics ability is 0.08 [0.07, 0.10], accounting for 33.89% of the total effect. The mediating effect of perceived teacher’s cognitive stimulation strategy-mathematics anxiety is the weakest, accounting for 4.60% of the total effect, and the mediating effect is 0.01 [0.01, 0.02]. Therefore, for those teachers who feel anxiety about mathematics, it will affect their decision-making ability.

**Table 3:** The results of chain intermediary analysis of students’ relationship and high-level mathematical ability.

Mediation	Effect value	Lower limit	Upper limit	Proportion of total effect(%)
Teacher–student relationship → perceiving teachers’ cognitive stimulation → high-level mathematical ability	0.1	0.09	0.12	35.98
Teacher–student relationship → mathematical anxiety → high-level mathematical ability	0.09	0.07	0.11	33.89
Teacher–student relationship → perceiving teachers’ cognitive stimulation → mathematics anxiety → high-level mathematics ability	0.01	0.01	0.02	4.6
Total indirect effect	0.2	0.18	0.22	74.48

### Sensitivity analysis of teacher–student relationship parameters

**Figure 1:**
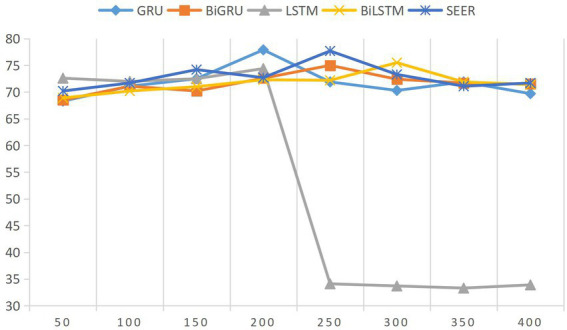
Influence of different hidden layer sizes on model results.

In this section, this paper will briefly discuss the influence of model efficiency, and visually show the influence results of different parameter values baseline method. The first discussion is about setting the size of the hidden layer. Setting the suitable size of the hidden layer is very important for the model effect. The suitable size of the hidden layer can better express the language text so as to extract the semantic information of the text more comprehensively. The influence of different data sizes on the results is shown in Figure 1. Through experiments, we find that all the teacher–student relationship models and the model proposed in this paper have a low impact on teacher–student relationship, except LSTM model. And when the size of the hidden layer is set to 250 dimensions, the effectiveness of this model reaches the highest. The influence of five main parameters (Bi-GRU layer number, hidden layer size, batch size, learning rate, and forgetting rate) on the efficiency of the model is briefly discussed, and the influence results of different parameter values on the deep learning model of baseline method and the model proposed in this paper are visually displayed.

The ordinate in Figure 1 represents a significant difference of 0.001 between the frequency and duration of interaction between teachers and students and the types of teacher–student relationship, which is between 0.1 and 0.2, indicating that the frequency and duration of interaction are weakly correlated with different types of teacher–student relationship. The larger the index, the more frequent the interaction. The ordinate represents the frequent number of interactions between students and teachers, and the higher the frequency, the higher the interaction with teachers and the better the relationship with teachers.

**Figure 2:**
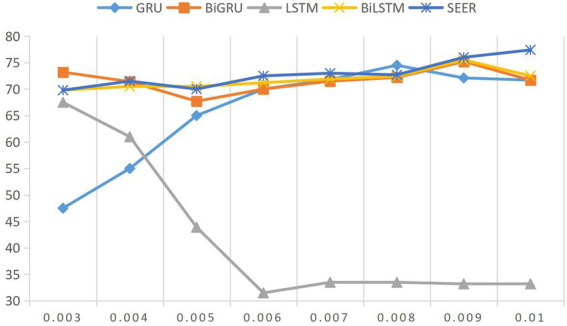
Influence of different learning rates on model results.

Next, we discuss the influence of parameter learning rate on the results of emotion model. Emotion analysis is based on the relationship between teachers and students. In this paper, the learning rate is distributed between 0.01 and 0.0001, and the experimental results are shown in Figure 2. Usually, if the learning rate is too high, the best solution may not be found, and if the setting value is too small, the training time will increase. Through experimental selection, 0.001 is finally selected as the learning average of this model.

**Figure 3:**
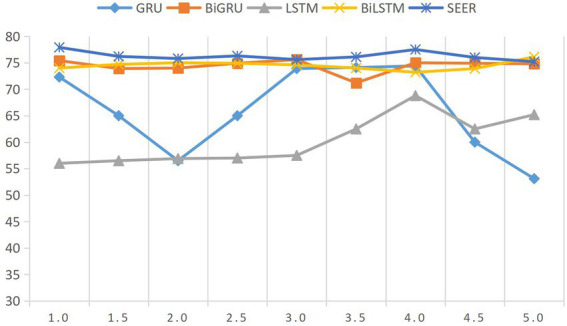
Influence of different Bi-GRU layers on model results.

Finally, the parameter discussed is the number of layers of Bi-GRU. When setting this parameter through experiments, it is found that unidirectional LSTM and unidirectional GRU are very sensitive to the value of network layers, but bidirectional LSTM and GRU have little influence, as shown in Figure 3. Considering the experimental environment of teacher–student relationship research, this paper chooses the case of the least experimental cost and sets the number of layers of Bi-GRU as one layer.

### Difference analysis of teacher–student interaction variables and teacher–student relationship types

**Figure 4:**
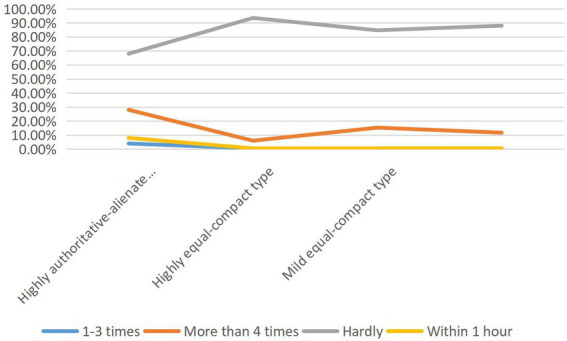
Cross-table Chi-square test of teacher–student interaction variables and teacher–student relationship types.

**Figure 5:**
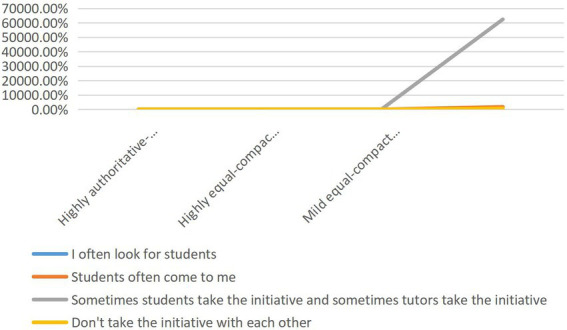
Cross table Chi-square test of teacher–student interaction variables and teacher–student relationship types.

The cross-chi-square test results of teacher–student interaction variables and teacher–student relationship types are shown in Figures 4, 5: there is no significant difference in interaction willingness among teacher–student relationship types (*p* = 0. 071 > 0.05). There was a significant difference between the frequency and duration of interaction and the type of teacher–student relationship at the level of 0.001 (*p* = 0. 000 < 0.001). Cramer’s V is between 0.1 and 0.2, which shows that the frequency and duration of interaction are weakly correlated with different types of teacher–student relationship. By comparing the A, B, C subscripts of the frequency of teacher–student relationship types, it is further concluded that there is no significant difference between “1–3 times of communication per semester” and “more than 4 times of communication” in highly authoritative-alienated type (a) and slightly equal-close type (a); There is no significant difference between the types (a) of teacher–student relationship in “the duration of each individual communication is more than 3 h.”

Combined with the percentage, in terms of interaction frequency, the proportion of tutors who communicate with graduate students 1–3 times per semester is significantly lower than that of mild equality-close type (15.3%) and highly authoritative-alienated type (28%). However, the proportion of tutors who communicate with graduate students more than 4 times per semester is significantly higher than that of mild equality-close type (84.7%) and highly authoritative-alienated type (68%). In terms of interaction duration, the teacher–student relationship of tutors who communicate with graduate students within 1 h each time is highly equal-close (20.1%) is significantly lower than that of mild equal-close (41.7%), but the teacher–student relationship of tutors who communicate with graduate students for 1–2 h and 2–3 h each time is highly equal-close (54.9%, 16.8%) is significantly higher than that of mild equal-close (44.3%, 9.3%). It can be seen that the effect of communication duration is obvious.

**Figure 6:**
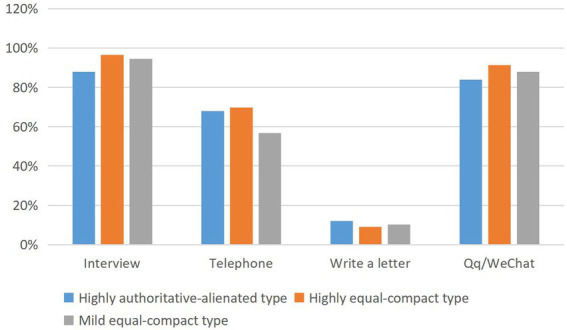
The main interaction modes between teachers and students and the types of teacher–student relationship.

In the way of interaction, “interview” is the most important way of interaction between tutors and graduate students in various types of teacher–student relations. “QQ/WeChat and other timely communication tools” followed closely. Moreover, the proportion of “interview” and “QQ/WeChat and other timely communication tools” in various types of teacher–student relationships is very close. One of the reasons may be that “QQ/WeChat and other timely communication tools” may still be the main way for teachers and students to make appointments and “interviews.” “Telephone” is the third main way for tutors to interact with graduate students after “interview” and “QQ/WeChat and other timely communication tools.” However, the proportion of “telephone” in highly equal-compact type (69.8%) was significantly higher than that in lightly equal-compact type (56.8%). The proportion of “writing letters” in all types of teacher–student relationships is relatively low, and there is no obvious difference (Figure 6).

To sum up, tutors who communicate with graduate students “more than 4 times per semester,” “each individual communication lasts between 1 and 3 h” and prefer to use “telephone” communication are closely related to the highly equal-close teacher–student relationship.

## Conclusion

Through the comparison of algorithm and experiment, it can be concluded that the teacher–student relationship affects the quality of teaching, and teachers should strive to improve their psychological quality and build an equal and democratic new teacher–student relationship. Equality, trust, understanding, tolerance and sincerity are the premise of the new teacher–student relationship. Teachers should treat students with the concept of equality, respect, understand and trust students, and influence and educate students with healthy personality, so that they become a generation of mental health. Good teaching process and teaching results will promote the emotional relationship between teachers and students to be more harmonious. Therefore, strengthening mutual understanding and communication between teachers and students is directly related to students’ learning and teachers’ teaching, and even has a great impact. Optimizing the psychological relationship between teachers and students is the realistic requirement of the reform of teacher–student relationship.

Teachers should respect students and treat students equally. Teaching should educate people first. Secondly, teachers should consolidate their own strength. Teachers’ own ability affects your prestige in students’ minds. Students should study hard to respect teachers and elders, so as not to contradict teachers.

## Data availability statement

The original contributions presented in the study are included in the article/supplementary material, further inquiries can be directed to the corresponding author.

## Author contributions

The author confirms being the sole contributor of this work and has approved it for publication.

## Funding

This work was supported by the phased achievements of the Anhui Provincial University ideological and political work ability improvement and the Provincial Construction Award and supplement project of the comprehensive reform pilot of “three integrity education” (sztsjh-2022-11-36) and the Anhui Medical University comprehensive reform pilot project of “three integrity education of “building a four in one ideological and political education model of classroom, campus, base and network” (2021xsqyr09).

## Conflict of interest

The author declares that the research was conducted in the absence of any commercial or financial relationships that could be construed as a potential conflict of interest.

## Publisher’s note

All claims expressed in this article are solely those of the authors and do not necessarily represent those of their affiliated organizations, or those of the publisher, the editors and the reviewers. Any product that may be evaluated in this article, or claim that may be made by its manufacturer, is not guaranteed or endorsed by the publisher.
